# ﻿A forgotten gem: *Mendonciaaffinis* (Acanthaceae), a new species once thriving at the Avenida Paulista, São Paulo, Brazil

**DOI:** 10.3897/phytokeys.260.153687

**Published:** 2025-07-11

**Authors:** Fabio Araújo da Silva, Cíntia Kameyama, Fabio A. Vitta, Silvana Buzato, Daniela C. Zappi

**Affiliations:** 1 Universidade Federal Rural da Amazônia/Museu Paraense Emílio Goeldi, Programa de Pós-Graduação em Ciências Biológicas - Botânica Tropical, Avenida Perimetral 1901, Terra Firme, Belém, PA, 66077–830, Brazil Universidade Federal Rural da Amazônia/Museu Paraense Emílio Goeldi Belém Brazil; 2 Herbário COOE – Instituto Federal de Rondônia, campus Colorado do Oeste, RO, BR 435, km 63, Zona Rural, 76.993–000, Brazil Instituto Federal de Rondônia Colorado do Oeste Brazil; 3 Herbário SP – Instituto de Pesquisas Ambientais, Av. Miguel Stéfano 3687, Água Funda, São Paulo, SP, 04301–902, Brazil Instituto de Pesquisas Ambientais São Paulo Brazil; 4 Departamento de Botânica, Universidade Estadual de Campinas, Campinas, SP, 13083–970, Brazil Universidade Estadual de Campinas Campinas Brazil; 5 Departamento de Ecologia, Universidade de São Paulo, São Paulo, SP, 05422–970, Brazil Universidade de São Paulo São Paulo Brazil; 6 Universidade Federal do Recôncavo da Bahia, CCAB, Rua Rui Barbosa 710, Cruz das Almas, BA, 44380–000, Brazil Universidade Federal do Recôncavo da Bahia Cruz das Almas Brazil; 7 Universidade de Brasília, Programa de Pós-Graduação em Botânica, Instituto de Ciências Biológicas, Caixa Postal 04457, Brasília, DF, 70910–970, Brazil Universidade de Brasília Brasília Brazil

**Keywords:** Atlantic Rainforest, Mendoncieae, new taxon, taxonomy, Thunbergioideae

## Abstract

Recent research towards a revision of Neotropical *Mendoncia* has confirmed the existence of a new species growing in the semideciduous seasonal forest (*mata de planalto*) component of the Atlantic Forest. As became evident from fieldwork carried out in Southeastern Brazil and following the study of numerous herbarium specimens, the new species is distinct from *M.velloziana*, from the coastal and septentrional ombrophilous forests, due to its golden-green bracteoles, densely pubescent ovary and puberulous fruits. A description of the new species is provided, along with a distribution map, conservation status assessment, illustrations and a comparative table with morphologically similar taxa.

## ﻿Introduction

The genus *Mendoncia* Velloso ex Vand. (Acanthaceae – Thunbergioideae) is currently placed in the tribe Mendoncieae (Manzitto-Tripp et al. 2022), and is characterized by climbers and lianas with flowers subtended by paired bracteoles and carnose, indehiscent fruits, found in tropical and subtropical areas. The center of diversity of the genus is the Amazon, where more than half of its 90 known species occur (Magnaghi and Daniel 2017). The Neotropical distribution of *Mendoncia* extends from southeastern Mexico to Central and South America, reaching Southern Brazil and Paraguay, while, in the Paleotropical region, the genus is represented by 14 species from tropical Africa and Madagascar (Magnaghi and Daniel 2014; Breteler and Wieringa 2018). Although the taxonomy and morphology of *Mendoncia* has received some attention (Nees 1847a, b; Turrill 1919; Leonard 1931, 1951; Profice 1988; Wasshausen and Wood 2004; Wasshausen 2006, 2013; Magnaghi and Daniel 2017; Breteler and Wieringa 2018; Silva et al. 2023; Flora and Funga of Brazil 2025), species delimitation in the Neotropical region is still challenging. In a floristic treatment for the Flora of São Paulo State, Buzato and Vitta (2005) suggested that a new taxon of *Mendoncia* from the more seasonal parts of the Atlantic Rainforest might need recognition. During the taxonomic revision of Neotropical *Mendoncia* (Silva et al. in prep.), this new species was confirmed, characterized by a unique combination of tomentose-velutinous to glabrescent, leaf-blades with brochidodromous venation, golden-greenish (*in vivo*), ovate to deltoid bracteoles, and densely pubescent to tomentose ovary.

This study aims to contribute to the knowledge of the genus *Mendoncia* by describing and illustrating this new species, as well as providing a comparative analysis of the morphological traits that distinguish it from closely related species, such as *Mendonciavelloziana* Mart. and *M.rosea* Leonard. This new discovery not only expands our understanding regarding the morphological diversity of the genus, but also provides insights for future research on the biogeography and conservation of neotropical plant species.

## ﻿Material and methods

In this study, specimens were examined in HUCP, IAN, INPA, MBM, MG, MO, R, RB, RON, SP, SPF, UEC, UPCB herbaria (acronyms according to Thiers 2025). The morphological terminology adopted to describe the shapes of leaf blades, bracteoles, flowers and fruits follow definitions by Radford et al. (1974), Harris and Harris (1994) and Magnaghi and Daniel (2017), while indumentum and trichomes follow Ahmad (1978), Payne (1978), Nogueira et al. (2013), and Silva et al. (2023).

The distribution map was created using QGIS 3.42.0 Brighton software (QGIS Development Team 2025), based on geographic coordinates obtained from herbarium labels.

The conservation assessment was carried out based on the IUCN criteria (IUCN 2012, 2024). GeoCAT (Bachman et al. 2011) was used to calculate Extension of Occurrence (EOO) and Area of Occurrence (AOO), the latter calculated by plotting known locality coordinates using a 2 km^2^ grid.

## ﻿Results

### ﻿Taxonomic treatment

#### ﻿ Mendoncia affinis

Taxon classificationPlantaeLamialesAcanthaceae

Vitta & Buzato sp. nov.

A3D2ADA0-428A-5638-B32E-03424E51EB90

urn:lsid:ipni.org:names:77365304-1

**Figure 1.**
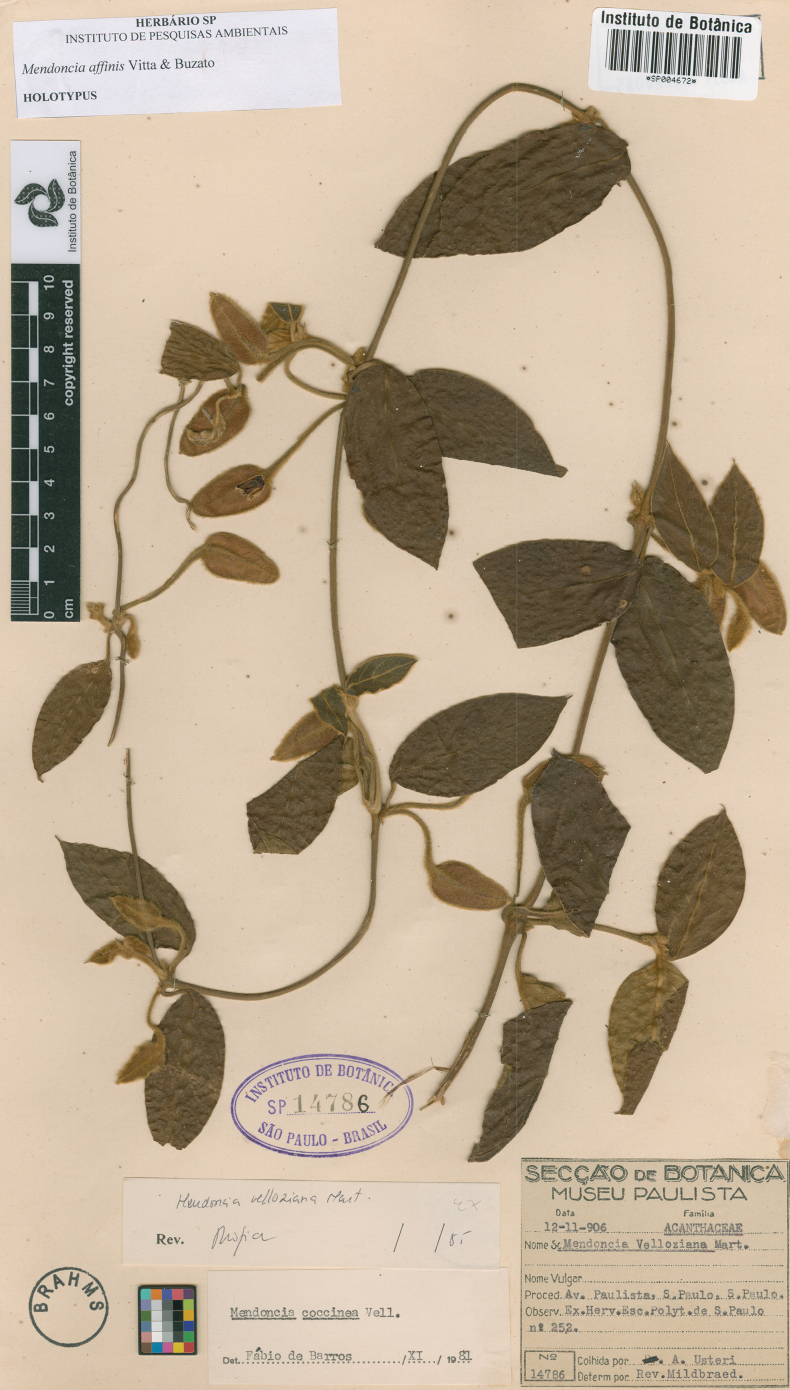
Image of the holotype of *Mendonciaaffinis* housed at SP herbarium.

**Figure 2.**
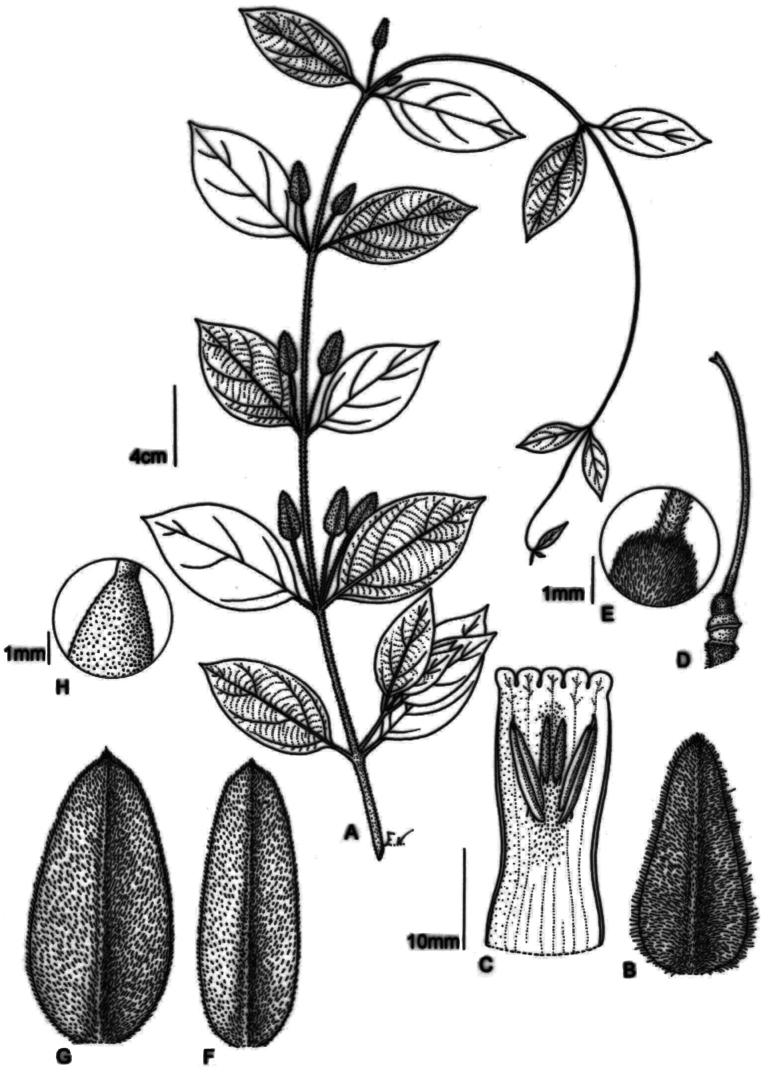
**A–E***Mendonciaaffinis*. **A.** Flowering branch; **B.** Bracteole; **C.** Open corolla; **D.** Gynoecium; **E.** Ovary detail; **F–H.***Mendonciavelloziana*; **F, G.** Bracteole; **H.** Ovary detail. Drawn by Emiko Naruto based on S. Buzato s.n. in UEC 20978 (A–E) and S. Buzato & A. Salino s.n. in UEC 28091 (F–H).

**Figure 3.**
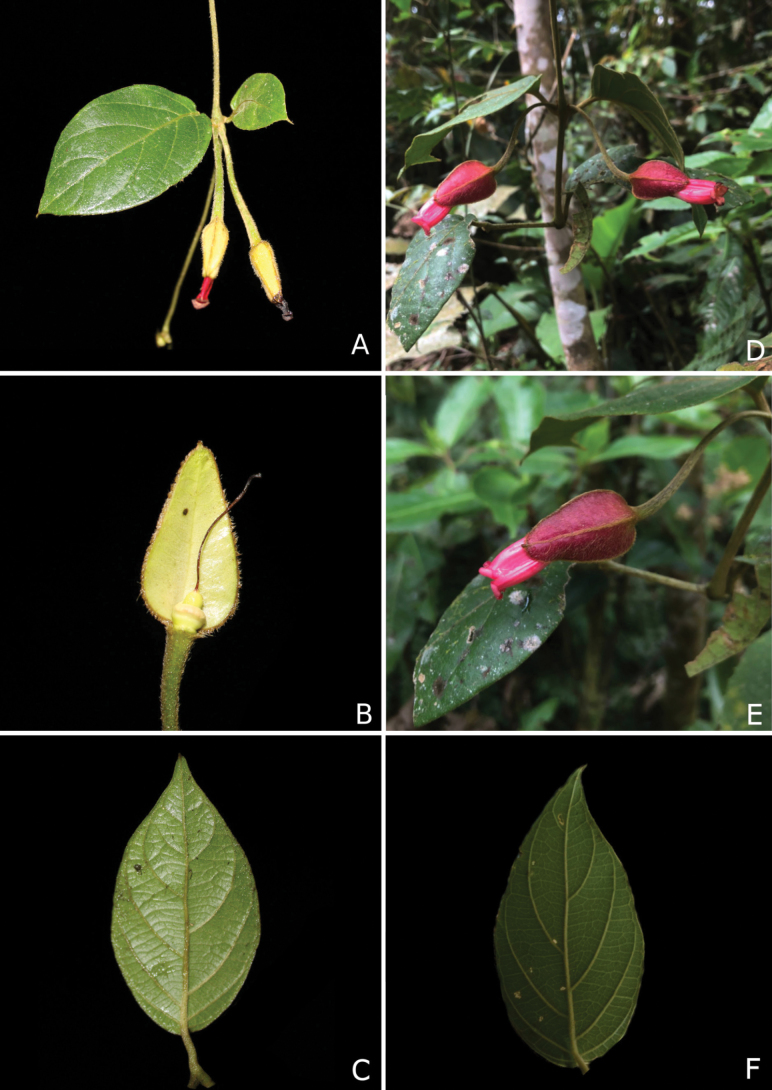
**A–C***Mendonciaaffinis*. **A.** Flowering branch; **B.** Details of the inner part of the bracteole and gynoecium; **C.** Leaf blade; **D–F.***Mendonciavelloziana*; **D.** Flowering branch; **E.** Flower detail; **F.** Leaf blade. Photos by D Nunes (A–C) and FA Silva (D–F).

Figs 1 , 2A–E , 3A–C

##### Type.

Brazil • São Paulo: Mun. São Paulo, Av. Paulista; 12 Nov. 1906, fl. and fr.; *A. Usteri 252* (holotype SP14786!).

##### Diagnosis.

*Mendonciaaffinis* differs from *M.velloziana* by the combination of subquadrangular to quadrangular, hollow, sulcate, tomentose-velutinous branches (vs. subcylindrical to subquadrangular, solid, sulcate, densely pubescent branches); petioles and pedicels with tomentose to densely pubescent indumentum, with adpressed to semi-erect eglandular hairs (vs. tomentose with adpressed eglandular hairs); bracteoles ovate to deltoid, golden-greenish (*in vivo*), 2–2.7 × 1–1.5 cm, glabrous internally (vs. ovate-lanceolate, red (*in vivo*), 2.4–3.3 × 1–1.6 cm, sparsely lepidote internally); leaf venation brochidodromous with 3–4 pairs of secondary veins (vs. eucamptodromous with 3–5 pairs); ovary densely pubescent to tomentose (vs. lepidote with glandular-peltate trichomes); style basally pilose and glabrous distally (vs. entirely glabrous); and oblongoid, pubescent drupes (vs. ellipsoid, glabrous drupes).

##### Description.

Herbaceous climbers, stems subquadrangular to quadrangular, fistulose, longitudinally sulcate when young, tomentose-velutinous to glabrescent, eglandular trichomes adpressed, golden, internodes 4.5–11.5 cm long. ***Leaves*** membranaceous; petiole 5–12 mm long, slightly canaliculated, tomentose to densely pubescent, eglandular trichomes adpressed to semi-erect; leaf-blade 5–10.5 × 2.5–5.3 cm, ovate-lanceolate to elliptic, base obtuse to cuneate, margin entire, apex acuminate to cuspidate, with mucro 0.9–1 mm long; venation brochidodromous, 3–4 pairs of secondary veins, midrib and secondary veins prominent, densely covered in eglandular trichomes; adaxial face smooth, glabrescent to sparsely strigose, eglandular trichomes semi-erect, abaxial side tomentose-velutinous, eglandular trichomes semi-erect to erect. ***Flowers*** 1–3(–4) per axil; pedicels 19–50 mm long, densely tomentose-velutinous, trichomes eglandular, adpressed to semi-erect; bracteoles 2–2.7 × 1–1.5 cm, golden-greenish (*in vivo*), ovate to deltoid, fused up to the apical third, subcoriaceous, midvein slightly prominent, base cuneate to truncate, apex mucronate, with mucro 1.2–1.8 mm long, externally densely pubescent to velutinous, eglandular trichomes semi-erect, brown (*ex vivo*), 0.8–1.2 mm long, internally glabrous; calyx 1.2–1.5 × 3–3.2 mm, entire, slightly dentate at margin, glabrous; corolla red, 2.5–2.7 cm long, tubular, tube slightly curved, externally glabrous, sparsely glandular-papillose within, base of tube 0.7–0.9 cm wide, middle of tube 0.6–0.7 cm wide, apex of tube 0.9–1 cm wide, 5 lobes 0.29–0.3 × 0.18–0.2 cm, oblong, emarginate, all lobes reflexed; free part of filaments 2–2.5 mm long, dorsal anthers with thecae 0.85–0.9 cm long, ventral anthers with thecae 1–1.2 cm long; ovary 2.4–3 × 2–2.2 mm, densely pubescent to tomentose, eglandular trichomes erect; style 2–2.2 cm long, proximally pilose, distally glabrous, eglandular trichomes erect, stigma bilobed, lobes subequal, 0.5–0.6 mm long. ***Drupe*** (immature) 1.1–1.2 × 0.9–1 cm, oblongoid, pubescent, with erect to semi-erect eglandular trichomes.

##### Etymology.

The specific epithet *affinis* refers to the morphological similarity between the new species and *Mendonciavelloziana*. This similarity led to a taxonomic confusion, where specimens of *M.affinis* were often mistakenly identified as *M.velloziana*, causing *M.affinis* to remain hidden under that name for a long time.

##### Phenology.

Flowering and fruiting specimens were collected throughout the year, more often during November and December (corresponding to spring in Southeastern and Southern Brazil).

##### Distribution and habitat.

**Figure 4.**
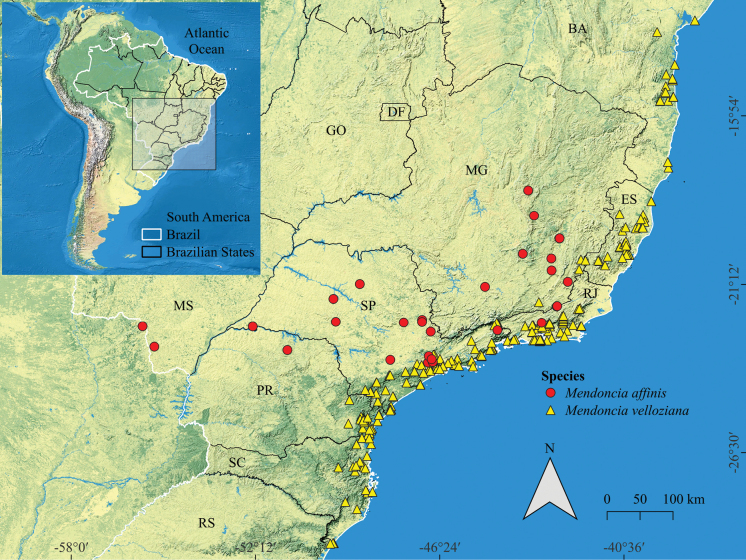
Map comparing the distribution of *Mendonciaaffinis* (red dots) and *M.velloziana* (yellow triangles). Abbreviations of the State: Bahia (BA), Distrito Federal (DF), Espírito Santo (ES), Goiás (GO), Mato Grosso do Sul (MS), Minas Gerais (MG), Paraná (PR), Rio de Janeiro (RJ), Rio Grande do Sul (RS), Santa Catarina (SC), São Paulo (SP).

*Mendonciaaffinis* occurs in Brazil and Paraguay, with a broad distribution across the semideciduous seasonal forests (*mata de planalto*) (Capobianco 2001) that extend from Southeastern and Southern Brazil into the bordering region of Paraguay. The species has been recorded in the Brazilian states of Minas Gerais, Rio de Janeiro, São Paulo, Paraná, Santa Catarina and Mato Grosso do Sul, and in the Department of Amambay in Paraguay (Fig. 4).

##### Conservation status.

The original distribution of *Mendonciaaffinis* comprised a wide area of semideciduous seasonal forest (Capobianco 2001) spanning from Minas Gerais and São Paulo to Amambay in Paraguay, reflecting an Extent of Occurrence of 443,000 km^2^. There are more than 50 collection localities for this species; however, the vast majority of the collections were made during the last century, and many of these localities are no longer extant due to urbanization (surroundings of São Paulo and Campinas) and landscape transformation (inner states of São Paulo, Paraná and Minas Gerais) (Ribeiro et al. 2009; Lima et al. 2020). In fact, considering the protection situation of the Atlantic Rainforest, the semideciduous seasonal forest has fewer protected sites than the perhumid ombrophilous forest, and is so severely fragmented that we considered the Area of Occupancy (AOO) of 116 km^2^ as an adequate measure of the distribution of the species, even though the original state of the forest was more or less continuous. This situation justifies the assessment of Endangered (EN) B2a,b(ii,iii,iv) attributed here to the species.

##### Additional specimens examined (paratypes).

**Brazil** • **Mato Grosso do Sul**: Amambaí, 1979, W.G. Garcia 13691 (UEC); • **Minas Gerais**: [Além Paraíba] Teixeira Soares, 01 fevereiro 1907, fl., A. Sampaio 673 (R); • [Além Paraíba] Teixeira Soares, Aug. 1907, fl., A. Sampaio s.n. (R45342); • [Itabirito] Itabira do Campo, 21 Nov. 1941, fr., M.G. Magalhães 753 (IAN); • Lavras, Reserva Biológica do Poço Bonito, 05 Dec. 1989, fr., R.J. Almeida & F.F. Avezum 187 (SPF); • Marliéria, Parque Florestal Estadual do Rio Doce, 19°47'0"S, 42°37'0"W, 18 May 1982, fl., H.P. Bautista 556 (MBM, MG, RB); • Marliéria, Reserva Florestal do Rio Doce, Matas da Lagoa Central, 30 Aug. 1973, fl., G. Martinelli & D. Sucre 26 (RB); • Muriaé, mata perto do córrego Barro Alegre, beira da BR 116, 23 outubro 1989, fr., J.R. Pirani et al. 2528 (SPF); • Ponte Nova, fazenda Varginha, 12 km, 07 Dec. 1958, fl. e fr., H.S. Irwin 2248 (R); Prox. de Conceição do Mato Dentro, 17 Feb. 1965, fl., A.P. Duarte 9100 (RB); • Diamantina, fazenda do Gavião, 21 Nov. 1937, fl., M. Barreto 9930 (R); • Viçosa, mata da Prefeitura, à 14 km do Inst. de Ciências Biol. da Universidade de Viçosa, 23 May 1978, fl., F.V. Vidal et al. 1050 (RB); • **Paraná**: Londrina, floresta dos Godoy, 10 Aug. 1985, fl., C. Chagas & F. Silva 853 (MBM); • **Rio de Janeiro**: Petrópolis, 1913, fr., A. Lutz 479 (R); • **São Paulo**: Mogi Guaçu, fazenda Campininha, mata da Mariana, córrego da Divisa, 04 Jun. 1991, fr., R. Neto & L. Rossi 1183 (SP); • Idem, 25 Sept. 1991, fl., S.R. Neto & R. Zifirino 1253 (SP); • São José do Barreiro, 25 Apr. 1894, fl., A. Loefgren 2466 (SP); • São Paulo, Jaraguá, 06 Jan. 1926, fr., F.C. Hoehne s.n. (SP); • Pindorama, propriedade Elidio Ribeiro, 07 Feb. 1939, fr., O.T. Mendes s.n. (IAN, SP44153); • Lins, Colonia Paraizo, 28 Jan. 1941, fl., G. Hashimoto 464 (SP); • Monte Alegre do Sul, Amparo, 26 Mar. 1943, fl., M. Kuhlmann 367 (SP); • São Paulo, Parque do Estado do São Paulo, 04 Apr. 1944, fl., W. Hoehne s.n. (SPF); Idem, 23 Nov. 1966, fl. e fr., R. Faria & O.G. Fonseca s.n. (SP); • Rio Claro, mata da Faz. São José, 25 Aug. 1984, fl., J. Brunini 162 (MBM, SPF); Itapetininga, 10 Dec. 1987, fr., A. Loefgren 443 (SP); • Gália, Reserva Ecológica de Caetetus, 12 Mar. 1989, fl., F.C. Passos 21040 (RB); São Paulo, Parque Santo Dias, trilha principal, 08 Dec. 1992, fr., R.J.F. Garcia 296 (SPF); • Teodoro Sampaio, Parque Estadual Morro do Diabo, 04 Jun. 1994, fl., V.S. Rodrigues 41 (SPF); • Campinas, Reserva Municipal da Fazenda de Santa Genebra, 20 Dec. 1988, fl., S. Buzato s.n. (UEC20978). **Paraguay** • **Amambay**: Sierra de Amambay, E. Hassler s.n. (MO, RB).

## ﻿Discussion and conclusions

The new species is primarily characterized by the tomentose-velutinous indumentum found on the stems and pedicels and the ovate to triangular golden-greenish sub-coriaceous bracteoles (Fig. 2A–E). *Mendonciaaffinis* was long included in *M.velloziana* due to the similarity in corolla colour and tomentose indumentum of several structures. Fieldwork in Southeastern Brazil, combined with the study of numerous herbarium collections, led to the recognition of this new taxon that appears to have a different distribution, occurring more inland, in seasonal semideciduous forest, while *M.velloziana* grows in perhumid, ombrophilous forests closer to the coast (Fig. 4). *Mendonciaaffinis* has ovate to triangular, golden-greenish bracteoles (*in vivo*; Fig. 3A, B) that are glabrous within. In contrast, *M.velloziana* has ovate-lanceolate, externally red bracteoles (*in vivo*; Fig. 3D, E) that are sparsely lepidote within. Additionally, *M.affinis* has a densely pubescent to tomentose ovary (vs. lepidote in *M.velloziana*) and pubescent, oblong drupes (vs. glabrous, ellipsoid drupes in *M.velloziana*). The new species also resembles *Mendonciarosea*, endemic to Colombia, primarily due to its membranous leaves, red corolla, and densely pubescent ovary. However, it differs by the tomentose-velutinous to glabrescent branches (vs. branches always pubescent in *M.rosea*), tomentose to densely pubescent petioles and pedicels (vs. densely sericeous petioles and pedicels in *M.rosea*), leaf blades adaxially smooth and brochidodromous (vs. leaf blades adaxially scabrous and eucamptodromous in *M.rosea*), glabrous calyx with a slightly dentate margin (vs. proximally pubescent calyx with repand margin in *M.rosea*), and proximally pilose style (vs. glabrous style in *M.rosea*) (Table 1).

**Table 1.** Morphological comparison of *Mendonciaaffinis* with similar species.

Character	*Mendonciaaffinis*	*Mendonciavelloziana*	*Mendonciarosea*
**Branches**	Subquadrangular to quadrangular, hollow, sulcate, tomentose-velutinous when young	Subcylindrical to subquadrangular, solid, sulcate, densely pubescent	Subcylindrical to subquadrangular, hollow, sulcate, pubescent
**Petiole and Pedicels**	Tomentose to densely pubescent, adpressed to semi-erect eglandular hairs	Tomentose, adpressed eglandular hairs	Densely sericeous, adpressed eglandular hairs
**Leaf blade base**	Obtuse to cuneate	Obtuse to acute	Obtuse to rounded
**Leaf blade (adaxial side)**	Glabrescent to sparsely strigose	Glabrescent to sparsely pubescent	Scabrous, puberulous
**Venation**	Brochidodromous, 3–4 pairs of secondary veins	Eucamptodromous, 3–5 pairs of secondary veins	Eucamptodromous, 3–4 pairs of secondary veins
**Bracteoles**	2–2.7 × 1–1.5 cm, golden-greenish, ovate to deltate, glabrous internally	2.4–3.3 × 1–1.6 cm, red, ovate-lanceolate, sparsely lepidote internally	2–2.2 × 1.2–1.3 cm, ovate, glabrous internally
**Calyx**	1.2–1.5 × 3–3.2 mm, glabrous	1.8–2 × 4–4.5 mm, glabrous	1.8–2 × 2.8–3 mm, basally pubescent, distally glabrous
**Ovary**	Densely pubescent to tomentose	Lepidote, glandular-peltate	Densely pubescent, adpressed eglandular hairs
**Style**	Basally pilose, glabrous distally	Glabrous	Glabrous
**Drupe**	oblongoid, pubescent	ellipsoidal, glabrous	ovoid, glabrous

The recognition of a new species that remained undiscovered in herbarium collections for more than 100 years highlights the historical importance of long standing, well-curated biological collections. An example of that is our choice of type-specimen, a very well preserved collection made back in 1907 in the now ‘iconic’ Avenida Paulista (Fig. 1), when this area was covered in Atlantic Rainforest. Nowadays only the Parque Siqueira Campos (Trianon) remains as a witness of what was once a natural environment. Through herbarium collections it is possible to know the full distribution of this and many other species, even if over 88% of the Atlantic Rainforest dominium is now lost or completely modified, with serious consequences both of biomass and biodiversity erosion (Ribeiro et al. 2009; Lima et al. 2020).

## Supplementary Material

XML Treatment for Mendoncia affinis

## ﻿Additional information

### Use of AI

No use of AI was reported.

### Author ORCIDs

Fabio Araújo da Silva https://orcid.org/0000-0002-1944-7246

Cíntia Kameyama https://orcid.org/0000-0001-6624-1884

Fabio A. Vitta https://orcid.org/0000-0002-8012-952X

Silvana Buzato https://orcid.org/0000-0002-9559-5152

Daniela C. Zappi https://orcid.org/0000-0001-6755-2238

### Data availability

All of the data that support the findings of this study are available in the main text.
